# Motivational Interviewing as a Strategy to Improve Adherence in IBD Treatment: An Integrative Review Amidst COVID-19 Disruptions

**DOI:** 10.3390/healthcare12121210

**Published:** 2024-06-18

**Authors:** Caterina Mercuri, Maria Catone, Vincenzo Bosco, Assunta Guillari, Teresa Rea, Patrizia Doldo, Silvio Simeone

**Affiliations:** 1 Clinical and Experimental Medicine Department, Magna Graecia University, 88100 Catanzaro, Italy; c.mercuri@unicz.it (C.M.); doldo@unicz.it (P.D.); silvio.simeone@unicz.it (S.S.); 2 Department of Public Health, University of Naples Federico II, 80138 Naples, Italy; maria.catone12@gmail.com (M.C.); teresa.rea@unina.it (T.R.); 3 Department of Medical and Surgical Sciences, University Hospital Mater Domini, Magna Graecia University, 88100 Catanzaro, Italy; vincenzo.bosco@unicz.it

**Keywords:** inflammatory bowel disease, motivational interviewing, therapeutic adherence, COVID-19, integrative review, patient engagement, telemedicine

## Abstract

**Aims and Objectives:** This review aims to analyze the effectiveness of motivational interviewing (MI) in enhancing therapeutic adherence and compliance in adult patients with inflammatory bowel disease (IBD), especially considering the disruptions caused by the COVID-19 pandemic. **Background:** IBD, which includes conditions such as ulcerative colitis and Crohn’s disease, affects over 10 million people globally. It significantly impacts both physical and psychological well-being, leading to challenges in therapeutic adherence. Only 25–47% of patients with IBD adequately follow prescribed treatments. **Design and Methods:** An integrative methodology that combines qualitative and quantitative research was utilized, following a 7-step framework. This framework encompasses identifying the research question, devising a search strategy, performing a critical appraisal, summarizing findings, extracting data, conducting an analysis, and drawing conclusions. **Results:** Poor adherence to therapy among patients with IBD can exacerbate disease progression and result in complications. MI has been identified as a promising approach to improving both adherence and treatment outcomes. Studies, including those predating the COVID-19 pandemic, have demonstrated MI’s effectiveness in enhancing adherence among patients with IBD. **Conclusions:** MI shows promise in enhancing adherence among adult patients with IBD. Although initial results are promising, additional research is needed to thoroughly understand its effectiveness across various clinical contexts. **Relevance to Clinical Practice:** The findings underscore the potential of MI as an integral component of IBD treatment strategies, suggesting that its implementation could enhance patient–provider interactions and lead to better overall health outcomes.

## 1. Introduction

Inflammatory bowel disease (IBD) is a common term for a series of clinical phenotypes caused by chronic, relapsing, and remitting inflammation of the gastrointestinal tract [1]. The main forms of IBD are represented by Crohn’s disease (CD) and ulcerative colitis (UC) [2], with UC involving continuous inflammation limited to the colon’s mucosa and CD characterized by transmural inflammation and skip lesions throughout the gastrointestinal tract [3]. IBD is a global disease involving more than 10 million people worldwide [4]. Epidemiological studies suggest the presence of approximately 3.2 million affected individuals in Europe, with over 2 million cases identified in North America, and millions more across the globe [5].

The main symptomatology of IBD includes abdominal pain, chronic and relapsing episodes, bloody diarrhea, nausea, vomiting, weight loss, anorexia, and fatigue [6]. Patients with IBD may also suffer from extra-intestinal manifestations involving the eyes (episcleritis), skin (erythemanodosum), and joints (peripheral and axial arthropathies) [7]. Furthermore, in some cases, patients with IBD may experience complications such as stenosis, fistulas, and infections [8] and present an increased risk of developing colon cancer [9]. Therefore, the complexity of this symptomatology can significantly compromise patients’ health and quality of life [10,11]. In fact, IBD affects life standards, leading to negative consequences on the ability of such patients to perform daily activities [12], often leading to feelings of shame, bodily dissatisfaction, and social isolation [13,14].

Despite advancements in therapy, there is no cure for IBD [15], and treatment focuses on inducing and maintaining remission, preventing complications, and improving quality of life [16,17,18].

Effective drug therapy for IBD requires precise adherence to treatment regimens [19]. However, patients may struggle with adherence due to fluctuating symptoms and perceptions of well-being during remission [20,21]. Poor adherence can contribute to disease progression and the development of short- and long-term complications [22]. Recent data suggest that a notable proportion of individuals diagnosed with IBD fail to adhere to prescribed medications, with non-adherence rates ranging from 53% to 75% [23]. Addressing medication adherence and treatment success in IBD requires a multifaceted approach [22] that includes interactive interventions with education and psychological support [20,21,22].

The relationship between healthcare providers and patients has always been the cornerstone of healthcare for patients with IBD [24], and motivational interviewing has emerged as a key strategy for improving therapeutic adherence [25]. The conceptual framework of motivational interviewing was originally described by William Miller [26]. Over the years, Miller and Rollnick have further developed and expanded the original concepts of motivational interviewing [27]. Motivational interviewing (MI) is a highly specified communicative approach that improves the patient–provider relationship and enables increased personal motivation for behavioral change interventions [28,29]. A systematic review of randomized controlled trials conducted in 2022 on motivational interviewing with adult patients suffering from chronic diseases revealed its supportive role in enhancing medication adherence [30].

Motivational interviewing is a tailored approach to addressing the unique concerns and barriers that individuals with IBD encounter in adhering to their treatment regimens, which is carried out within a non-stigmatizing and non-judgmental atmosphere of acceptance and compassion [31]. Through empathetic listening and collaborative goal-setting, motivational interviewing helps patients explore their ambivalence toward treatment [25]. Patients, indeed, may grapple with concerns about medication side effects, financial constraints, or the perceived inconvenience of treatment regimens [32]. Furthermore, motivational interviewing enables patients to actively participate in their care journey. Integrating motivational interviewing into clinical practice can improve patient outcomes and contribute to the delivery of patient-centered care in IBD management [28].

To our knowledge, few studies have investigated the impact of motivational interviewing on IBD adult patients. The last review on MI efficacy in patients with IBD was conducted in 2017. Its results highlighted MI’s effectiveness in both improving and sustaining adherence [33]. 

The COVID-19 outbreak, subsequently, has posed an unprecedented challenge to humanity and science [34], resulting in a significant and abrupt disruption of health services, disproportionately affecting people with chronic conditions including IBD [35]. The disruption of routine services has significantly affected individuals with chronic conditions, such as IBD, who rely on regular monitoring and treatment for maintaining optimal health and continuity with the same clinical team to prevent discontinuity of care [36]. Although one of the goals in managing chronic diseases is to promote self-management and self-efficacy, unprecedented events such as the COVID-19 pandemic disrupted some patients’ ability to be self-determined in managing their inflammatory bowel disease, causing some frustration [36]. 

Specifically, patients experienced interruptions or reduced adherence to therapies, often due to difficulties in contacting physicians or healthcare facilities and to a shortage of medications [37]. In this context, measures implemented in response to the pandemic have limited patients’ ability to access healthcare facilities and research institutes [38]. As a result, telemedicine, during the COVID-19 pandemic, became crucial for stable patients with IBD not requiring infusion therapy, presenting an opportunity to integrate MI into remote care modalities [39]. In particular, telemedicine enabled the continuity of care and remote monitoring [40].

Therefore, the COVID-19 pandemic has highlighted the importance of adapting the delivered care to the needs of patients with IBD. The restrictions and conditions imposed by the pandemic situation have made clear the need for innovative and flexible solutions to ensure effective management of the disease. The COVID-19 pandemic has underscored the importance of adapting care modalities to meet patients’ evolving needs, emphasizing the role of innovative solutions like MI in promoting therapeutic adherence and patient engagement. In light of the COVID-19 pandemic’s disruption to healthcare services and its impact on chronic illnesses like IBD, this integrative review seeks to explore how motivational interviewing (MI) could enhance treatment adherence and patient engagement under these new challenges. This study endeavors to fill a significant gap in the current research, providing comprehensive insights into the incorporation and potential advantages of MI within IBD treatment protocols. Ultimately, the goal is to contribute towards the enhancement of IBD management strategies, thereby improving patient engagement and health outcomes.

## 2. Materials and Methods

The current article is an integrative review that aims to analyze the results of various scientific research studies conducted through different study designs [41,42]. This review adheres to the seven-step conceptual framework proposed by Dhollande et al. (2021) [42], which includes (1) identifying the research question; (2) determining the search strategy; (3) critically appraising the results; (4) summarizing research findings; (5) extracting data; (6) analyzing; and (7) drawing conclusions and discussing implications. Integrative reviews offer several advantages, such as evaluating the quality of included studies and combining results from both qualitative and quantitative literature [43]. Therefore, they are considered valuable in healthcare decision-making, providing a comprehensive synthesis of available literature and facilitating the effective utilization of its findings [44,45,46].

### 2.1. Identification of the Research Question 

The PIO (Population, Intervention, Outcome) method was utilized to develop the research question for this integrative review (Table 1). The population of interest comprises adults diagnosed with inflammatory bowel diseases (IBDs), specifically ulcerative colitis and Crohn’s disease. The intervention under investigation is motivational interviewing, which is examined without a comparison group. The primary outcome of interest is the patient’s adherence and/or compliance with their therapeutic regimen, encompassing medication intake, lifestyle adjustments, and other prescribed treatment components.

Therefore, after applying the PIO method, the research question that this review aims to answer is as follows:

RQ1: *Can Motivational Interviewing improve therapeutic adherence and/or compliance in adult patients with IBD?*

### 2.2. Search Strategy

The search was conducted on CINAHL Complete, MEDLINE, APA PsycInfo, APA PsycArticles, and Psychology and Behavioral Sciences Collection databases. The search string was structured by combining identified keywords such as “Inflammatory Bowel Disease,” “Motivational Interviewing,” “Counseling,” and/or “Counseling” through the Boolean operators “AND” and “OR,” resulting in the following: “(“motivational interviewing” OR “counseling” OR “counseling”) AND “inflammatory bowel disease*”. The only filter applied to the search strategy was “adult” given the paucity of data in the literature about this specific topic. The search string formulation emerged through collaborative efforts among expert reviewers. Subsequently, all authors collaborated in the retrieval of articles and engaged in comprehensive readings of relevant full text pertaining to the search question. 

### 2.3. Inclusion and Exclusion Criteria

Integrative reviews synthesize both qualitative and quantitative research, offering a comprehensive understanding of the subject under investigation [43,47]. In line with literature recommendations, this review also considers systematic reviews and meta-analyses to ensure a robust and inclusive analysis of existing evidence [44]. The inclusion criteria for this review are as follows: (a) scientific articles of any study design, including, but not limited to, randomized controlled trials, observational studies, and case studies; (b) studies focusing on the adult population; (c) research investigating motivational interviewing and its impact on the compliance/adherence of patients with inflammatory bowel disease (IBD). The exclusion criteria include (a) duplicate publications; (b) studies focusing on the pediatric population; (c) non-English articles and literature reviews without original data (if applicable); and (d) studies not directly exploring the application of motivational interviewing in IBD.

## 3. Results

### 3.1. Study Selection

**Figure 1:**
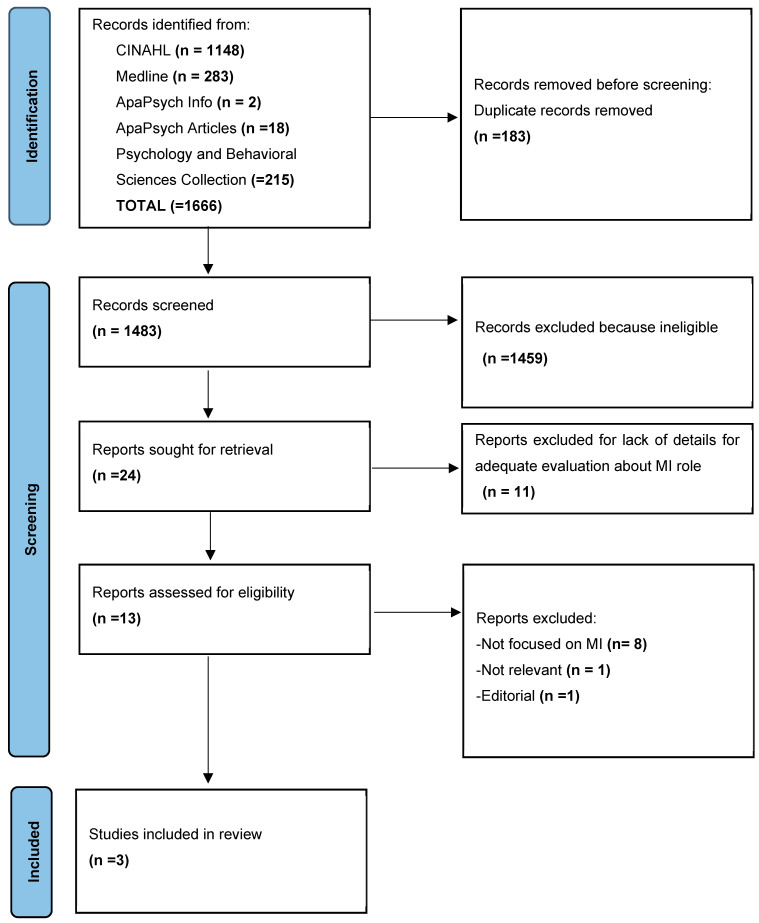
PRISMA flow diagram [48].

This integrative review was conducted between November 2023 and February 2024. The search strategy provided 1666 results as follows: 1148 from CINAHL Complete, 283 from Medline, 2 from APA PsycInfo, 18 from APA PsycArticles, and 215 from Psychology and Behavioral Sciences Collection. A total of 183 duplicates were removed leaving 1483 articles for screening based on reading title and abstract. From these, a total of 1459 articles were excluded because they were deemed irrelevant to the research objective. Of the remaining 24 articles, 13 were included for full-text analysis. This left 13 articles whose full text was downloaded and read by two independent reviewers. This step led to the exclusion of 10 articles because they were not consistent with the inclusion criteria; specifically, 8 of these did not focus on motivational interviewing, 1 of these was a letter to the editor and 1 was not relevant. At the end of the process, 3 articles were included. This integrative review was conducted independently by two reviewers with the supervision of two experts in case discrepancies arose. The detailed process of article selection is shown in Figure 1 based on the PRISMA (Preferred Reporting Items for Systematic Reviews and Meta-Analysis) model [48]. 

### 3.2. Quality Appraisal

The evaluation of the methodological quality of the included studies was conducted through the utilization of the QuADS Tool (Quality Assessment with Diverse Studies). This tool, selected for its reliability and validity across various study designs such as quantitative, qualitative, mixed, and multimethod studies, encompasses 13 evaluation criteria scored from 0 (lowest) to 3 (highest) as detailed in Table 2 [49,50]. To ensure rigorous evaluation, two independent reviewers conducted the quality assessment, resolving any scoring discrepancies through discussion. The maximum attainable score for each study was capped at 39, representing the sum of the maximum scores across all criteria. The final score for each study was determined as a percentage, calculated by the ratio of the total score to the total criteria score [final score = total score of each study/total criteria score × 100%] [50].

### 3.3. Key Characteristics of Included Studies

The main characteristics of the included studies are summarized in Table 3. Thus, a total of three scientific papers were included in this review. Two of these are case reports, while one is a systematic review summarizing the results of four papers, specifically two randomized controlled trials (RCTs) and two quasi-experimental studies. The evaluation of the methodological quality of the included studies identified two works of medium–high quality, whose values are 92% and 51% [33,51], while the remaining one is characterized by a value of 33%, so it was evaluated as low-quality [52].

**Table 2:** Quality Assessment with Diverse Studies (QuADS) scores for each paper.

Authors—Year	Theoretical or Conceptual Underpinning to the Research	Statement of Research Aim/s	Clear Description of Research Setting and Target Population	The Study Design is Appropriate to Address the Stated Research Aim/s	Appropriate Sampling to Address the Research Aim/s	Rationale for Choice of Data Collection Tool/s	The Format and Content of Data Collection Tool is Appropriate to Address the Stated Research Aim/s	Description of Data Collection Procedure	Recruitment Data Provided	Justification for Analytic Method Selected	The Method of Analysis was Appropriate to Answer the Research Aim/s	Evidence that the Research Stakeholders Have been Considered in Research Design or Conduct	Strengths and Limitations Critically Discussed	Total Score (%)
Ramdeen et al., 2014 [52]	1	1	2	3	2	0	1	1	0	0	1	0	1	13 (33%)
Wagoner and Kavookjan, 2017 [33]	3	3	3	3	3	3	3	3	3	3	3	0	3	36 (92%)
Antal-Uram, Harsányi and Perczel-Forintos, 2018 [51]	3	3	1	3	2	1	3	1	0	1	2	0	0	20 (51%)

**Table 3:** Data extraction of included articles.

Authors—Year	Summary of Findings	References
Ramdeen et al., 2014 [52]	This case report involves a 27-year-old Caucasian man diagnosed with Crohn’s disease and shows the use of MI in a nonconfrontational manner to increase cooperation and motivation for health-related changes. While the single case report does not demonstrate the method’s effectiveness, a comprehensive understanding of the theories behind MI can empower nurses and physicians to apply this technique in referral settings.	[52]
Wagoner and Kavookjan, 2017 [33]	This systematic review includes four articles, comprising two randomized controlled trials (RCTs) and two quasi-experimental studies, with a total sample size ranging from 45 to 278 patients aged between 20 and 82 years. Motivational interviewing demonstrates effectiveness in improving health outcomes, particularly in terms of adherence, help-seeking behavior, and perceptions about empathy from healthcare providers, in patients with IBD. Strengths of the study include its comprehensive review of available literature on MI and patients with IBD. However, limitations include the lack of exclusively RCTs. The findings suggest that healthcare providers may benefit from utilizing MI to enhance patient–provider relationships and communication skills, thereby improving patient outcomes in IBD management.	[33]
Antal-Uram, Harsányi, and Perczel-Forintos, 2018 [51]	This case report examines the role of a psychologist in managing a 21-year-old patient with Crohn’s disease who also presents with psychiatric disorders, including mood dysregulation and avoidant personality disorder. The intervention options explored include low-intensity cognitive behavioral therapy, including motivational interviewing. The results indicate that psychotherapy sessions incorporating motivational interviewing have led to the remission of mental health symptoms, improved drug adherence, and enhanced quality of life for the patient. Recognizing and addressing psychiatric comorbidities can significantly improve adherence to drug treatment and overall quality of life. Interdisciplinary collaboration is essential to ensure a holistic approach to patient care, encompassing biological, psychological, and spiritual dimensions.	[51]

### 3.4. Results of the Included Studies 

The case report by Ramdeen et al. (2014) describes the use of motivational interviewing with a 27-year-old patient with Crohn’s disease. Through an account of an interaction between a nurse and the patient, the paper aims to clarify the principles that guide practitioners in this approach. In particular, it explains a strategy for conducting non-confrontational discussions between healthcare providers and patients, emphasizing that a thorough understanding of the underlying theories facilitates the use of this technique to address common problems associated with the disease [52]. The case report by Antal-Uram et al. (2018), written in Hungarian, examines the case of a 21-year-old individual struggling with psychiatric conditions and Crohn’s disease. This case involves the introduction of low-intensity psychotherapy sessions incorporating motivational interviewing. The aim is to determine the impact of mood disorders and Crohn’s disease-related symptoms on treatment adherence and whether social withdrawal is due to physical symptoms or reflects intrinsic personality traits. Results suggest that the treatment of psychiatric comorbidities improves adherence and quality of life by correcting maladaptive interpretations of the disease. Furthermore, the integration of motivational interviewing into low-intensity psychotherapy emerges as a viable method to achieve these goals [51]. Finally, the systematic review by Wagoner and Kavookjan (2017) shows that patients with inflammatory bowel disease (IBD) respond positively to motivational interviewing. In fact, this approach may influence patient perceptions and satisfaction with the quality of care because of the positive impact of motivational interviewing on provider–patient communication and relationships [33]. In particular, additional findings from specific studies within the paper show that, in addition to improving adherence [53,54], motivational interviewing can implement adherence to follow-up visits and smoking cessation [31] and has a positive impact on quality of life and information-seeking [55].

## 4. Discussions

This integrative review was conducted to explore the effectiveness of motivational interviewing (MI) in improving therapeutic adherence and compliance in adult patients with inflammatory bowel disease (IBD). The IBD population is at a higher risk of reduced quality of life due to the physical and psychological consequences of the disease [14,56]. In fact, the course of the pathology, fluctuating between remission and exacerbation, leads to poor therapeutic adherence (with non-adherence rates between 53% and 75%) [23]. The results of the included studies indicate that the use of motivational interviewing is an effective strategy to optimize therapeutic adherence in patients with IBD. 

These results may stem from the active involvement of the patient during motivational interviewing. Specifically, MI enhances the patient’s awareness and understanding of their health conditions [57], thereby improving participation in healthcare plans proposed by healthcare professionals and resulting in better health outcomes [58].

In fact, in the included case report by Antal-Uram et al. (2018), the adoption of low-intensity cognitive behavioral therapy, including motivational interviewing, resulted in an increase in therapeutic adherence from 20% to 75%, leading to disease regression and a reduction in the number of medications taken [51].

More broadly, this study underscores the importance of addressing patients’ psychological well-being. Conditions like depression and anxiety are prevalent among patients with IBD and profoundly affect both adherence to treatment and overall quality of life [59,60]. Therefore, the use of behavioral change strategies is a possible application to manage both psychological and somatic symptoms [51,61]. In particular, it is necessary to tailor the treatment to the needs of the patient [51]. In this context, motivational interviewing has shown advantages related to its adaptability and short-term goals that allow for continuous patient follow-up. This flexibility includes the potential to use remote strategies to overcome disease-related barriers and to monitor patients for long periods, even in remission [62]. As underscored in the clinical case reported by Ramdeen et al. (2014), grasping the technique of motivational interviewing by healthcare professionals and its application in the patient relationship can lead to more effective management of IBD [52].

In particular, strengthening the patient–doctor relationship is a key step in improving adherence, addressing thoughts that impact concrete actions, and eliciting behavioral changes with positive outcomes [52,63]. 

The systematic review by Wagoner and Kavookjan (2017) suggests that the implementation of motivational interviewing through individual meetings and telephone interviews had a positive consequence on adherence and patient perception concerning the relationship with the healthcare professional, making them more inclined to seek advice regarding their health [33].

Employing MI techniques can lead healthcare providers to engage patients in meaningful conversations about their treatment regimens and can collaboratively develop strategies to overcome barriers impacting adherence and/or compliance [33]. By embracing a patient-centered approach, healthcare providers can create a supportive environment that empowers patients to take an active role in their care and enhance their confidence in managing their condition [64].

However, these results need to be interpreted in light of the current healthcare landscape, which has been significantly impacted by the COVID-19 pandemic [65]. The COVID-19 pandemic has caused unprecedented disruptions in various aspects of gastroenterology healthcare services worldwide [66], emphasizing the need to modify the delivery methods of healthcare services [67] to implement a proactive approach to care. The health services most affected were those dealing with the management of chronic diseases [68].

The management of chronically ill patients was greatly affected by the pandemic, given the significant mental and physical impact on their behaviors, attitudes, and perceptions [66,69,70,71]. This has resulted in an ongoing cycle of stress, depression, and disease activity relapse [24], along with notably diminished adherence to prescribed medications [24,72]. 

Two studies have shown that a significant number of patients with IBD have delayed or canceled hospital visits due to fear of contracting COVID-19 [71,73]. In Lebanon, over 50% of patients with IBD in the study delayed hospital treatment [71], while in Korea, more than half of the patients surveyed canceled or postponed their hospital visits [73]. However, telemedicine and phone consultations have emerged as viable solutions to ensure that patients with IBD receive the care they need [71,74]. Patients have shown a high level of satisfaction with these approaches [71]. Telemedicine, in particular, has proven to be an optimal solution during the COVID-19 pandemic and has highlighted new therapeutic approaches centered around counseling and motivational interviewing techniques to improve treatment adherence in individuals with IBD [23,75].

Notably, the literature suggests that MI can be implemented in remote care and can be effective in various patient populations, responding to the diverse needs of those receiving assistance [76,77,78]. Therefore, the results emerging from our review highlight the importance of promoting motivational interviewing in a chronic condition such as IBD, where poor patient compliance can lead to worsening of the disease, increased healthcare costs and morbidity, and a reduction in quality of life [79].

The adaptability of MI to different approaches, including remote ones, makes it a therapeutic option with the potential to maintain high-quality care for patients with IBD; moreover, it should be implemented in light of the disease characteristics and the modifications in healthcare services caused by the pandemic [31,80].

Despite the experience of the COVID-19 pandemic and the high percentage of non-adherence in patients with IBD [81], motivational interviewing has not been exhaustively explored in the scientific literature. The COVID-19 pandemic has significantly impacted the publication priorities within the biomedical scientific community [82]. 

The quantity and quality of data available for analysis are limited, representing the main limitation of our article. Additional weaknesses depend on the variability of the samples involved in the included studies, heterogeneity in the conduct of motivational interviewing (number of sessions, adopted approach, and duration of meetings), and variability in the methodologies used to assess adherence. Additionally, the included studies did not specify the disease phase of the patient and whether professionals were trained in conducting motivational interviewing. Therefore, it is appropriate to conduct further studies to explore the technique and application of motivational interviewing in patients with IBD, in different contexts and for different treatment regimens. 

## 5. Conclusions

In conclusion, this integrative review has highlighted the impact of motivational interviewing (MI) on adherence and/or compliance in adult patients with inflammatory bowel disease (IBD), particularly within the context of the COVID-19 pandemic. Our findings indicate that MI could serve as a valuable behavioral intervention to enhance patient engagement in disease management. However, it is crucial to underscore that the existing literature in this domain warrants further investigation. 

## Figures and Tables

**Table 1:** PIO structure.

Population	Adults with inflammatory bowel disease (IBD)
Intervention	Motivational interviewing
Outcome	Improvement of therapeutic adherence or compliance

## Data Availability

Data are contained within the article.
